# SGLT2 inhibition, acylcarnitines and heart failure: a Mendelian randomization study

**DOI:** 10.1136/openhrt-2024-003078

**Published:** 2025-09-01

**Authors:** Zhiyu Wu, Shuyao Song, Jun Lv, Canqing Yu, Dianjianyi Sun, Pei Pei, Ling Yang, Yiping Chen, Iona Y Millwood, Robin G Walters, Hong Guo, Xiaoming Yang, Dan Schmidt, Junshi Chen, Zhengming Chen, Liming Li, Yuanjie Pang

**Affiliations:** 1 Department of Epidemiology & Biostatistics, Peking University School of Public Health, Beijing, China; 2 Key Laboratory of Epidemiology of Major Diseases (Peking University), Ministry of Education, Beijing, China; 3 Clinical Trial Service Unit & Epidemiological Studies Unit (CTSU), University of Oxford Nuffield Department of Population Health, Oxford, England, UK; 4 Liuyang Traditional Chinese Medical Hospital, Liuyang, China; 5 China National Center for Food Safety Risk Assessment, Beijing, China

**Keywords:** Epidemiology, Genetics, Heart Failure

## Abstract

**Objective:**

Sodium-glucose cotransporter 2 (SGLT2) inhibitors are guideline-recommended agents for treating heart failure (HF), but the role of metabolomic biomarkers in underlying mechanisms, particularly acylcarnitines, remains unclear. This study examined the associations of acylcarnitines with SGLT2 inhibition and incident HF.

**Methods:**

This subcohort study included 2178 participants from the prospective China Kadoorie Biobank without cardiovascular disease, diabetes or cancer at baseline. Plasma levels of 40 acylcarnitines were quantified using targeted mass spectrometry-based platforms. The impact of genetically predicted random plasma glucose (RPG) via SGLT2 inhibition on acylcarnitines was assessed with Mendelian randomization (MR). The associations of acylcarnitines with HF risk were assessed using Cox proportional hazards models. Acylcarnitines were classified into short-, medium- and long-chain groups and analysed individually or summed as scores.

**Results:**

Of the 2178 participants, the mean (SD) age was 53.2 (9.8) years. 13 incident HF cases occurred during a median follow-up of 10.5 years. SGLT2 inhibition was associated with higher levels of acylcarnitines, while higher levels of acylcarnitines were associated with reduced HF risk. An unweighted acylcarnitines score was associated with SGLT2 inhibition (β, 2.04 (0.29, 3.79) SD increase per 1 mmol/L lower genetic RPG via SGLT2 inhibition) and HF risk (HR, 0.97 (0.93, 0.99) per 1-SD higher of the score). Glucokinase activation, another antidiabetic agent used for comparison, showed weaker associations with acylcarnitines.

**Conclusion:**

MR analysis indicated SGLT2 inhibition showed associations with acylcarnitines, which are also associated with HF risk. Our findings highlighted the potential involvement of acylcarnitines in the mechanisms between SGLT2 inhibitors and HF.

**WHAT IS KNOWN ALREADY ON THIS TOPIC**

- Sodium-glucose cotransporter 2 (SGLT2) inhibitors are guideline-recommended agents for heart failure (HF) treatment.
- Acylcarnitines have been shown to be associated with both risk of diabetes and HF, but there is little evidence on the role of acylcarnitines in the underlying mechanisms.

**WHAT THIS STUDY ADDS**

- In a cohort study of 2178 individuals in China, lower levels of genetically predicted random plasma glucose via SGLT2 inhibition showed associations with higher levels of acylcarnitines, which were associated with a lower risk of HF.

**HOW THIS STUDY MIGHT AFFECT RESEARCH, PRACTICE OR POLICY**

- Awareness of the indispensable role of acylcarnitines in the effects of SGLT2 inhibitors on HF and provide support for further mechanism exploration.

## Introduction

 Sodium-glucose cotransporter 2 (SGLT2) inhibitors have emerged as an antidiabetic agent with additional cardiovascular benefits, especially in heart failure (HF).^1^ The broad metabolic improvements induced by SGLT2 inhibitors have not been fully elucidated, which may be the underlying mechanisms of the cardioprotective effect. Metabolomics could provide insights into the pathways of SGLT2 inhibitors on HF. As important metabolomic biomarkers involved in cellular energy metabolism pathways, acylcarnitines are considered as early biomarkers of type 2 diabetes^2^ and are associated with risk of HF.^3^ However, the role of acylcarnitines in the effect of SGLT2 inhibitors on HF has not been investigated.

Given the cost and ethical issues of clinical trials, drug-target Mendelian randomization (MR) provides an approach to simulate the effects of medicines on disease outcomes leveraging randomly allocated genetic variants located in the target genes.^4^ To investigate whether the cardioprotective effect of SGLT2 inhibition on HF was mediated by acylcarnitines, we performed a drug-target MR analysis to assess the causal effect of SGLT2 inhibition on circulating acylcarnitines and further examined the association between acylcarnitines and incident HF. We hypothesised that this cardioprotective effect may be specific to SGLT2 inhibition. To highlight this specificity, another antidiabetic agent, glucokinase activator, was selected for comparison.

## Methods

### Study populations and measures

China Kadoorie Biobank (CKB) is a large-scale population-based cohort recruiting 512 714 adults aged 30–79 years from 10 geographically diverse regions in China during 2004–2008, with three periodical resurveys involving ~5% participants.^5^ Participant information was collected through standardized questionnaires and physical examinations in baseline and non-fasting blood samples were collected for genotyping and an immediate on-site test of random plasma glucose (RPG). Genotyping information was available for 100 706 participants.^6^

**Figure 1:**
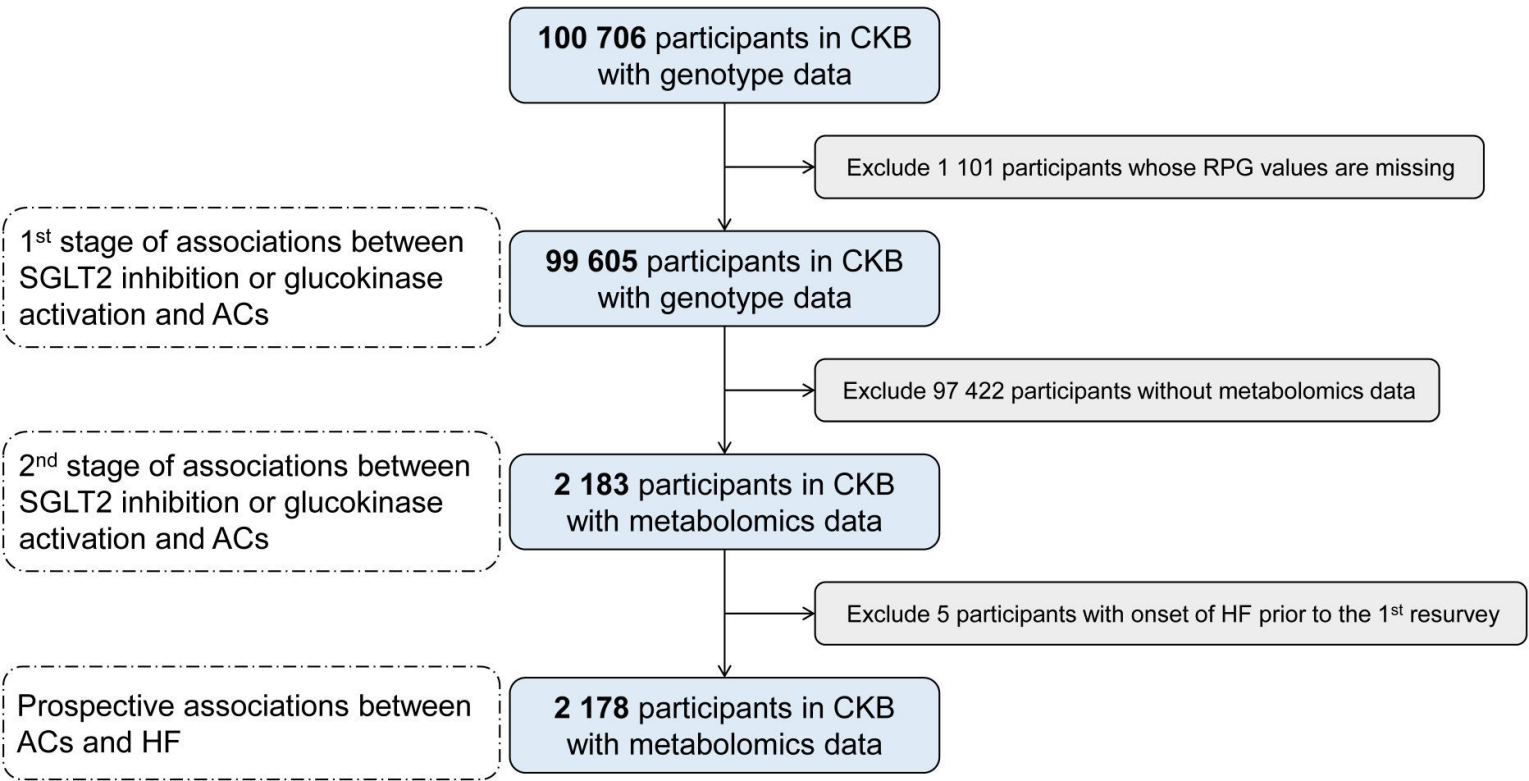
Flow chart of the analysis. AC, acylcarnitine; CKB, China Kadoorie Biobank; HF, heart failure; RPG, random plasma glucose; SGLT2, sodium-glucose cotransporter 2.

This study involved participants attending the first re-survey (considered the ‘baseline’ of the current study) of CKB who met the following criteria: (1) No baseline history or medication of cardiovascular disease, diabetes or cancers; (2) Time since last ate ≥8 hours; (3) With genotyping information (figure 1). Plasma levels of 40 acylcarnitines were quantified with targeted metabolomics platforms, using tandem mass spectrometry with the Biocrates MxP Quant 500 Kit. Incident cases of HF were identified by electronic linkage to death and disease registries and national health insurance claims databases (the International Classification of Diseases, 10th revision (ICD-10): I50), via a unique personal identification number.

### Genetic Risk Score for SGLT2 inhibition

We selected two genetic variants (online supplemental table 1) to proxy the hypoglycaemic effect of SGLT2 inhibitors following three steps. First, a genome-wide association study was conducted to identify variants associated with RPG located in gene *SLC5A2* (the target gene of SGLT2) with a p-value<0.05 (BOLT-LMM software, adjusting for age, sex, regions, fasting time and 11 national principal components). Second, a standard clumping process was performed to remove variants in high linkage disequilibrium (r^2^>0.2). Third, a Polygenic Risk Score (PRS) was constructed by calculating a weighted sum of the number of RPG-decreasing alleles. F-statistics were calculated to assess instrument strength and an F-statistic above 10 was considered as sufficient for the present study.

### Statistical analysis

Raw values of all acylcarnitines were log-transformed and then standardised prior to the analysis. Acylcarnitines were divided into short-chain (C2–C5), medium-chain (C6–C12) and long-chain (C13–C20) groups. Preprocessed levels of acylcarnitine with p<0.1 for association with SGLT2 inhibition or HF were transformed into the direction of increasing RPG according to results of individual acylcarnitine analyses and then summed to obtain unweighted acylcarnitine scores.

We first evaluated the effect of SGLT2 inhibition on acylcarnitines via RPG using two-stage least squares MR analysis adjusting for age, sex, regions, fasting time, antidiabetic agent therapy, array type and 11 national principal components. RPG was regressed on PRS in participants with genomic data (first stage, n=99 605, figure 1), and the fitted values were subsequently used as independent variables in regression with acylcarnitines (second stage, n=2183, figure 1). We then assessed the associations of acylcarnitines with incident HF risk in participants without HF (n=2178, figure 1) using Cox proportional hazards models, adjusting for sex, age, regions, smoking status and alcohol consumption.

For glucokinase activation, we selected seven genetic variants to conduct a PRS (online supplemental table 1) and repeated the above analyses.

## Results

Among all 2178 participants, the mean (SD) age was 53.2 (9.8) years, and 65.2% were women. During a median follow-up of 10.5 (IQR (10.4–10.5)) years, 13 incident HF cases were recorded. Participants who developed HF were older at baseline compared with non-cases (p<0.001), while no significant differences were observed in other baseline characteristics. Detailed baseline characteristics of all participants are shown in online supplemental table 2.

In MR analysis, the F-statistics of PRS for SGLT2 inhibition and glucokinase activation were 13.17 and 181.75, respectively. Of all 40 acylcarnitines, genetically predicted lower RPG via SGLT2 inhibition was significantly associated with higher levels of three acylcarnitines (C5:1, C10 and C14:1, figure 2a, online supplemental table 3), while genetically predicted lower RPG via glucokinase activation was only associated with one acylcarnitine (C14:1, figure 2b, online supplemental table 3). C14:1 showed association with genetically predicted lower RPG via both SGLT2 inhibition and glucokinase activation, with similar strength of effect (β (95% CI), −0.20 (−0.36,–0.04) and −0.20 (−0.35, –0.04), respectively).

**Figure 2:**
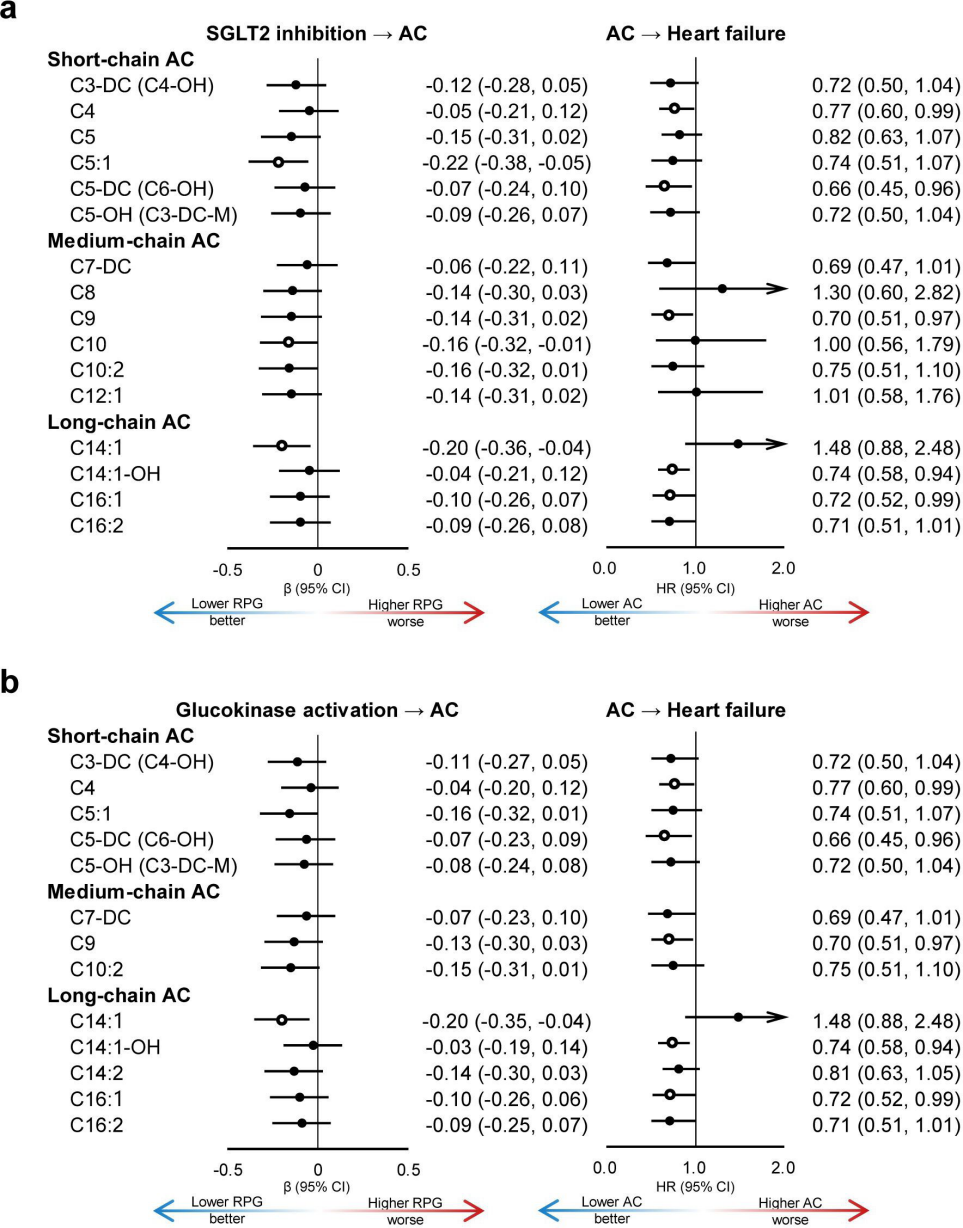
Association of SGLT2 inhibition, glucokinase activation with AC and heart failure. Forest plots of associations of AC with (**a**) SGLT2 inhibition, (**b**) glucokinase activation and heart failure. Open circles indicated that the associations were significant (p<0.05). ACs were divided into three groups: short-chain (**C2–C5**), medium-chain (**C6–C12**) and long-chain (**C13–C20**). Associations between SGLT2 inhibition and ACs were evaluated using Mendelian randomization analysis, reporting the SD change per 1-mmol/L lower genetically predicted RPG via SGLT2 inhibition. Associations between ACs and heart failure were evaluated using Cox proportional hazards models, reporting the HR of heart failure per 1-SD higher of AC or AC score levels. The threshold for statistical significance was set at p<0.05 if not otherwise specified. Raw value of p was reported due to the correlations of ACs and exploratory nature of the study. AC, acylcarnitine; RPG, random plasma glucose; SGLT2, sodium-glucose cotransporter 2.

In the Cox model, higher levels of five acylcarnitines were associated with lower risk of HF, including C4, C5-DC (C6-OH), C9, C14:1-OH, C16:1 (figure 2a, online supplemental table 3). Among them, C9 showed marginally significant associations with SGLT2 inhibition (p=0.091).

**Figure 3:**
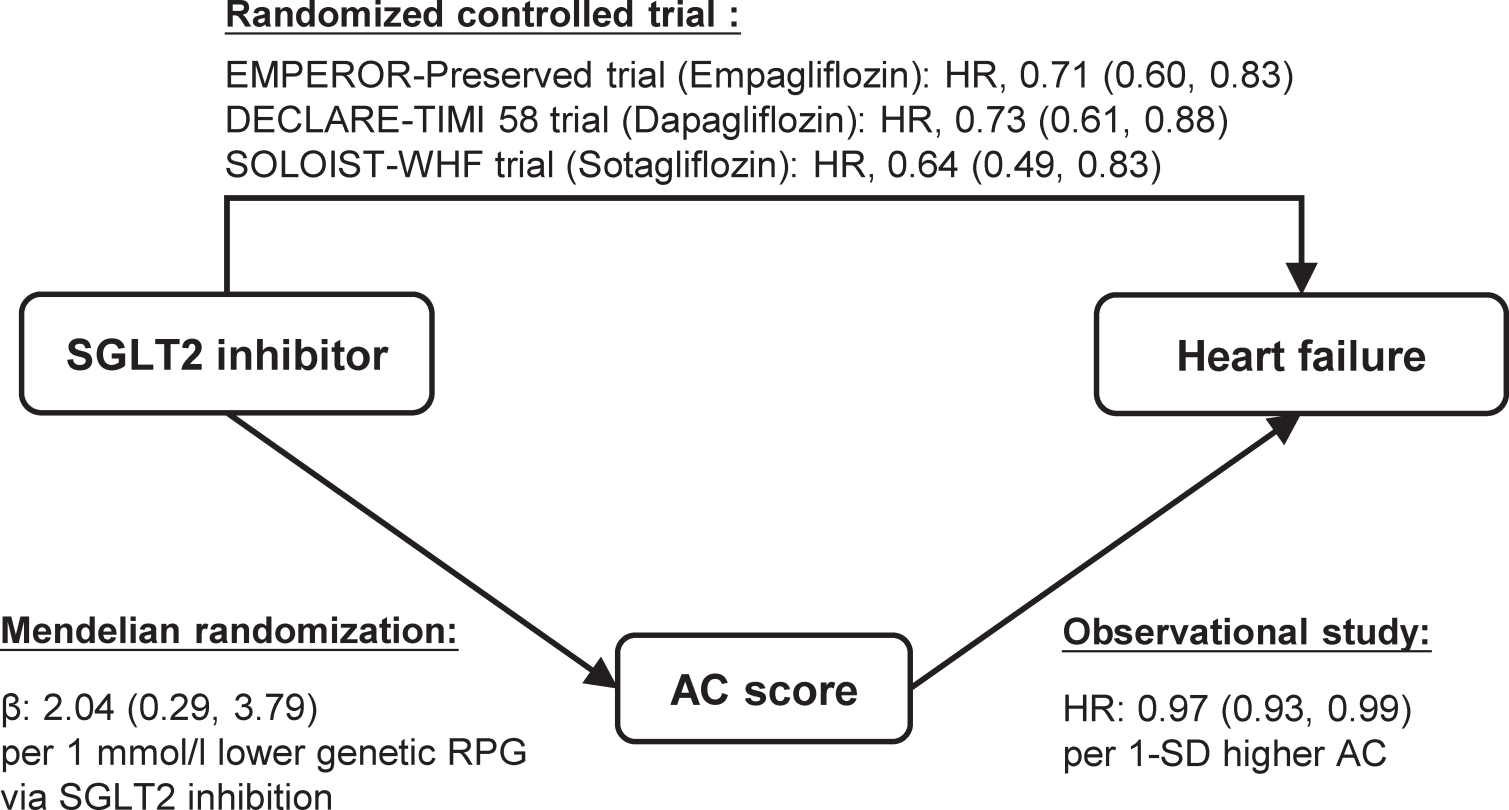
Associations of SGLT2 inhibition, AC score and heart failure. AC, acylcarnitine; DECLARE–TIMI 58, Dapagliflozin Effect on Cardiovascular Events–Thrombolysis in Myocardial Infarction 58; EMPEROR-Preserved, Empagliflozin Outcome Trial in Patients with Chronic Heart Failure with Preserved Ejection Fraction; RPG, random plasma glucose; SGLT2, sodium-glucose cotransporter 2; SOLOIST-WHF, Effect of Sotagliflozin on Cardiovascular Events in Patients with Type 2 Diabetes Post Worsening Heart Failure.

When constructed into an unweighted score, acylcarnitines were associated with both SGLT2 inhibition and HF (figure 3, online supplemental table 4). Our results showed that SGLT2 inhibition was associated with 2.04 (95% CI 0.29, 3.79) SD increase in acylcarnitines score per 1-mmol/L lower genetic RPG via SGLT2 inhibition, and per 1-SD higher score was associated with 3% lower risk of HF (HR (95% CI), 0.97 (0.93, 0.99)). SGLT2 inhibition was also associated with higher scores of medium-chain acylcarnitines (β (95% CI), −0.80 (−1.45,–0.16)), and short-chain acylcarnitines were significantly associated with HF (HR (95% CI), 0.92 (0.86, 0.98)). Compared with SGLT2 inhibition, glucokinase activation showed weaker associations with acylcarnitines (online supplemental table 5). Lower RPG level via genetic glucokinase activation was marginally associated with higher overall acylcarnitines scores (p=0.068). Overall, short-chain and medium-chain acylcarnitine scores were all associated with risk of HF (HR (95% CI), 0.96 (0.93, 0.99), 0.90 (0.83, 0.98) and 0.86 (0.74, 0.99), respectively).

## Discussion

In the present study, we showed that genetic inhibition of SGLT2, as well as genetic activation of glucokinase, was associated with acylcarnitines, which were associated with lower risk of HF.

Previous randomized controlled trials (RCTs) have demonstrated the cardioprotective benefits of SGLT2 inhibitors on HF (figure 3).^7^,^9^ Recently, several MR studies were conducted to explore the molecular mechanisms involved. For instance, the mediation effect of inflammatory biomarkers in the associations between SGLT2 inhibitors and HF was evaluated in a two-sample MR study.^10^ Moreover, several studies have explored the role of metabolites in the cardioprotective effects of SGLT2 inhibition, such as atrial fibrillation^11^ and cerebral small vessel disease,^12^ and found the mediating effects of the total concentration of lipoprotein particles and concentration of high-density lipoprotein particles on atrial fibrillation, 4-acetamidobutanoate on small vessel stroke, and the cholesterol to oleoyl-linoleoyl-glycerol ratio on the radial diffusivity of white matter. These studies evaluated the potential effects of multiple metabolites in a hypothesis-free approach, the biological plausibility of which remains to be explored. Our study extended the literature by focusing on the specific effect of acylcarnitines and also constructed scores to evaluate the overall effect.

There is limited evidence on the alterations in acylcarnitines induced by SGLT2 inhibitors. In a post hoc analysis of an RCT (n=234), an increase in short-chain and medium-chain acylcarnitines was observed after a 12-week treatment of SGLT2 inhibitors,^13^ which was also found in our study. Previous studies have assessed the prognostic value of acylcarnitines in patients with HF and found that long-chain acylcarnitines were associated with adverse clinical outcomes,^14^ while short-chain acylcarnitines showed inconsistent associations.^15^ Besides, a cross-sectional study (n=98) reported higher levels of acylcarnitines in patients with HF,^3^ which was inconsistent with our findings.

With a 10-year follow-up, our findings could avoid reverse causality and reflect long-term predictive value of acylcarnitines on HF rather than altered metabolic levels after the onset of HF. Future studies are warranted to investigate the benefits of SGLT2 inhibitors beyond their glucose-lowering effects. Functional validations, such as colocalisation analysis and druggability evaluation, are essential to investigate underlying mechanisms among SGLT2 inhibition, acylcarnitines and HF. Moreover, evidence for effects of glucokinase activators on acylcarnitines is lacking and needs to be explored.

Our study had several limitations. First, the sample size was limited and the genetic instrument to proxy the hypoglycaemic effect of SGLT2 inhibitors had modest power. Nonetheless, we selected glucokinase activation with stronger genetic instruments than SGLT2 for comparison and obtained reliable associations with acylcarnitines. Second, our study measured 40 acylcarnitines using a targeted platform and did not cover the whole spectrum of acylcarnitines. Future research using an untargeted platform or assessing more metabolic biomarkers on related pathways is warranted. Experimental studies or functional analyses are needed to elucidate the molecular mechanisms by which acylcarnitines may mediate the cardiovascular benefits of SGLT2 inhibition. Third, information on incident HF was obtained based on the ICD-10 codes, and echocardiographic data were not available, limiting our ability to distinguish between HF subtypes. Future studies incorporating imaging data are needed to further explore the differential effects of SGLT2 inhibition and acylcarnitines on specific HF subtypes.

In conclusion, our study in a Chinese population showed that acylcarnitines were associated with both SGLT2 inhibition and incident HF risk. The findings may provide insights into the underlying mechanisms linking SGLT2 inhibitors and HF.

## Supplementary material

10.1136/openhrt-2024-003078 online supplemental file 1

## Data Availability

Data are available in a public, open access repository.
