# Supplementary material for: SGLT2 inhibition, acylcarnitines and heart failure: a Mendelian randomization study
Source: Open Heart. 2025 Sep 1;12(2):e003078. doi: 10.1136/openhrt-2024-003078 (PMC12406923; doi:10.1136/openhrt-2024-003078)
Supplement: online supplemental file 1 [file openhrt-12-2-s001.docx]

**Supplementary Table 1. Genetic variants selected to conduct PRS for SGLT2 inhibition and glucokinase activation**

| **SNP** | **Chr** | **Pos** | **EA** | ***P*-value** |
| --- | --- | --- | --- | --- |
| **SGLT2 inhibition** | | | | |
| rs8057326 | 16 | 31524123 | T | 6.27E-03 |
| rs145874043 | 16 | 31497152 | C | 6.39E-03 |
| **Glucokinase activation** | | | | |
| rs2908289 | 7 | 44223942 | A | 2.69E-35 |
| rs3757840 | 7 | 44231216 | T | 7.77E-28 |
| rs59374739 | 7 | 44266570 | G | 4.87E-14 |
| rs6971410 | 7 | 44191190 | T | 1.42E-11 |
| rs887686 | 7 | 44185888 | C | 1.44E-10 |
| rs2284769 | 7 | 44222220 | C | 3.37E-10 |
| rs2908290 | 7 | 44216137 | A | 3.28E-08 |

**Supplementary Table 2. Baseline characteristics of participants**

| **Variable** | **All**  **(N=2178)** | **Case**  **(N=13)** | **Non-case**  **(N=2165)** | **P** |
| --- | --- | --- | --- | --- |
| **Sociodemographic characteristic** |  |  |  |  |
| Age, year | 53.2 ± 9.8 | 66.2 | 53.1 | **<0.001** |
| Female, % | 1419 (65.2) | 69.9 | 65.1 | 0.692 |
| Urban, % | 596 (27.4) | 30.2 | 27.3 | 0.853 |
| Middle school and above, % | 821 (37.7) | 34.9 | 37.7 | 0.713 |
| **Fasting time, h** | 13.3 ± 1.6 | 13.7 | 13.3 | 0.263 |
| **Lifestyle factors** |  |  |  |  |
| Current smoking^a^, % | 508 (23.3) | 26.4 | 23.3 | 0.771 |
| Weekly drinking, % | 286 (13.1) | 18.7 | 13.1 | 0.503 |
| Total physical activity, MET-h/day | 25.9 ± 15.7 | 24.1 | 25.9 | 0.827 |
| Dietary habits |  |  |  |  |
| Days consumed fresh vegetables /week | 6.7 ± 0.9 | 6.7 | 6.7 | 0.960 |
| Days consumed fresh fruits /week | 2.5 ± 2.4 | 2.2 | 2.5 | 0.492 |
| Days consumed red meat /week | 3.1 ± 2.3 | 3.3 | 3.1 | 0.745 |
| Adiposity |  |  |  |  |
| BMI, kg/m^2^ | 23.1 ± 3.3 | 24.1 | 23.1 | 0.314 |
| Waist circumference, cm | 78.4 ± 9.6 | 84.1 | 78.4 | 0.062 |
| **Family history** |  |  |  |  |
| Heart attack | 53 (2.4) | 0.0 | 2.5 | 0.996 |
| Stroke | 305 (14.1) | 0.0 | 14.1 | 0.991 |

Abbreviations: MET, metabolic equivalent of task; BMI, body mass index.

The results were presented as means or percentages adjusted for age, sex, and region, if applicable.

^a^Former smoker who had stopped smoking due to illness was classified as current smoker.

**Supplementary Table 3. Associations of** **acylcarnitines with SGLT2 inhibition, glucokinase activation, and heart failure**

| **Acylcarnitine** | **SGLT2 inhibition**  **→ Acylcarnitine** | |  | **Glucokinase activation → Acylcarnitine** | |  | **Acylcarnitine**  **→ Heart failure** | |
| --- | --- | --- | --- | --- | --- | --- | --- | --- |
|  | **β (95% CI)** | ***P*** |  | **β (95% CI)** | ***P*** |  | **HR (95% CI)** | ***P*** |
| C0 | -0.02 (-0.19, 0.15) | 0.815 |  | 0.02 (-0.14, 0.18) | 0.790 |  | 1.46 (0.45, 4.75) | 0.531 |
| **Short-chain acylcarnitine** | | | | | | | | |
| C2 | -0.13 (-0.29, 0.04) | 0.131 |  | -0.09 (-0.25, 0.07) | 0.275 |  | 1.49 (0.81, 2.76) | 0.201 |
| C3 | -0.05 (-0.21, 0.11) | 0.551 |  | -0.04 (-0.20, 0.11) | 0.582 |  | 1.05 (0.66, 1.69) | 0.830 |
| C3:1 | -0.09 (-0.26, 0.07) | 0.275 |  | -0.08 (-0.24, 0.09) | 0.357 |  | 0.79 (0.53, 1.18) | 0.253 |
| C3-DC  (C4-OH) | -0.12 (-0.28, 0.05) | 0.168 |  | -0.11 (-0.27, 0.05) | 0.173 |  | 0.72 (0.50, 1.04) | 0.083 |
| C3-OH | -0.01 (-0.16, 0.14) | 0.904 |  | -0.03 (-0.18, 0.12) | 0.719 |  | 0.73 (0.41, 1.31) | 0.289 |
| C4 | -0.05 (-0.21, 0.12) | 0.573 |  | -0.04 (-0.20, 0.12) | 0.609 |  | 0.77 (0.60, 0.99) | **0.043** |
| C4:1 | -0.06 (-0.22, 0.11) | 0.491 |  | -0.05 (-0.22, 0.11) | 0.512 |  | 0.85 (0.52, 1.41) | 0.534 |
| C5 | -0.15 (-0.31, 0.02) | 0.084 |  | -0.13 (-0.29, 0.04) | 0.131 |  | 0.82 (0.63, 1.07) | 0.142 |
| C5:1 | -0.22 (-0.38, -0.05) | **0.010** |  | -0.16 (-0.32, 0.01) | 0.052 |  | 0.74 (0.51, 1.07) | 0.113 |
| C5:1-DC | -0.06 (-0.23, 0.10) | 0.448 |  | -0.08 (-0.24, 0.09) | 0.365 |  | 1.08 (0.59, 1.95) | 0.809 |
| C5-DC  (C6-OH) | -0.07 (-0.24, 0.10) | 0.398 |  | -0.07 (-0.23, 0.09) | 0.413 |  | 0.66 (0.45, 0.96) | **0.030** |
| C5-M-DC | -0.03 (-0.20, 0.13) | 0.687 |  | -0.04 (-0.21, 0.12) | 0.594 |  | 0.88 (0.54, 1.45) | 0.615 |
| C5-OH  (C3-DC-M) | -0.09 (-0.26, 0.07) | 0.278 |  | -0.08 (-0.24, 0.08) | 0.337 |  | 0.72 (0.50, 1.04) | 0.085 |
| **Medium-chain acylcarnitine** | | | | | | | | |
| C6:1 | -0.02 (-0.19, 0.14) | 0.772 |  | -0.01 (-0.18, 0.15) | 0.872 |  | 0.74 (0.51, 1.08) | 0.117 |
| C6  (C4:1-DC) | -0.12 (-0.29, 0.04) | 0.146 |  | -0.12 (-0.28, 0.04) | 0.155 |  | 0.89 (0.60, 1.31) | 0.554 |
| C7-DC | -0.06 (-0.22, 0.11) | 0.496 |  | -0.07 (-0.23, 0.10) | 0.422 |  | 0.69 (0.47, 1.01) | 0.055 |
| C8 | -0.14 (-0.30, 0.03) | 0.099 |  | -0.11 (-0.27, 0.05) | 0.170 |  | 1.30 (0.60, 2.82) | 0.502 |
| C9 | -0.14 (-0.31, 0.02) | 0.091 |  | -0.13 (-0.30, 0.03) | 0.106 |  | 0.70 (0.51, 0.97) | **0.032** |
| C10 | -0.16 (-0.32, -0.01) | **0.045** |  | -0.12 (-0.27, 0.04) | 0.136 |  | 1.00 (0.56, 1.79) | 0.998 |
| C10:1 | -0.08 (-0.25, 0.08) | 0.316 |  | -0.08 (-0.24, 0.08) | 0.336 |  | 1.39 (0.79, 2.46) | 0.254 |
| C10:2 | -0.16 (-0.32, 0.01) | 0.060 |  | -0.15 (-0.31, 0.01) | 0.066 |  | 0.75 (0.51, 1.10) | 0.141 |
| C12 | 0.13 (-0.04, 0.29) | 0.133 |  | 0.13 (-0.03, 0.29) | 0.125 |  | 1.19 (0.63, 2.28) | 0.589 |
| C12:1 | -0.14 (-0.31, 0.02) | 0.092 |  | -0.11 (-0.27, 0.05) | 0.187 |  | 1.01 (0.58, 1.76) | 0.963 |
| C12-DC | 0.04 (-0.13, 0.20) | 0.676 |  | 0.06 (-0.10, 0.22) | 0.454 |  | 1.10 (0.67, 1.79) | 0.710 |
| **Long-chain acylcarnitine** | | | | | | | | |
| C14 | -0.11 (-0.28, 0.06) | 0.197 |  | -0.11 (-0.27, 0.05) | 0.182 |  | 1.10 (0.59, 2.04) | 0.758 |
| C14:1 | -0.20 (-0.36, -0.04) | **0.014** |  | -0.20 (-0.35, -0.04) | **0.012** |  | 1.48 (0.88, 2.48) | 0.137 |
| C14:1-OH | -0.04 (-0.21, 0.12) | 0.606 |  | -0.03 (-0.19, 0.14) | 0.747 |  | 0.74 (0.58, 0.94) | **0.014** |
| C14:2 | -0.12 (-0.29, 0.05) | 0.157 |  | -0.14 (-0.30, 0.03) | 0.099 |  | 0.81 (0.63, 1.05) | 0.110 |
| C14:2-OH | -0.06 (-0.22, 0.11) | 0.515 |  | -0.06 (-0.22, 0.11) | 0.486 |  | 0.78 (0.55, 1.10) | 0.161 |
| C16 | -0.06 (-0.22, 0.11) | 0.488 |  | 0.00 (-0.16, 0.16) | 0.967 |  | 1.13 (0.72, 1.76) | 0.599 |
| C16:1 | -0.10 (-0.26, 0.07) | 0.254 |  | -0.10 (-0.26, 0.06) | 0.224 |  | 0.72 (0.52, 0.99) | **0.049** |
| C16:1-OH | -0.04 (-0.21, 0.13) | 0.632 |  | -0.04 (-0.20, 0.13) | 0.658 |  | 0.75 (0.52, 1.08) | 0.125 |
| C16:2 | -0.09 (-0.26, 0.08) | 0.279 |  | -0.09 (-0.25, 0.07) | 0.272 |  | 0.71 (0.51, 1.01) | 0.052 |
| C16:2-OH | -0.08 (-0.25, 0.09) | 0.339 |  | -0.08 (-0.25, 0.08) | 0.322 |  | 0.78 (0.54, 1.15) | 0.212 |
| C16-OH | -0.10 (-0.26, 0.07) | 0.244 |  | -0.09 (-0.25, 0.07) | 0.258 |  | 1.19 (0.68, 2.08) | 0.543 |
| C18 | -0.06 (-0.23, 0.10) | 0.439 |  | -0.04 (-0.20, 0.12) | 0.658 |  | 1.00 (0.56, 1.77) | 0.994 |
| C18:1 | -0.06 (-0.22, 0.11) | 0.498 |  | -0.02 (-0.18, 0.13) | 0.760 |  | 1.19 (0.79, 1.79) | 0.403 |
| C18:1-OH | -0.11 (-0.28, 0.05) | 0.189 |  | -0.08 (-0.24, 0.08) | 0.309 |  | 1.12 (0.60, 2.09) | 0.730 |
| C18:2 | -0.02 (-0.18, 0.14) | 0.778 |  | 0.00 (-0.15, 0.15) | 0.999 |  | 0.97 (0.56, 1.67) | 0.898 |

*Associations of acylcarnitines with SGLT2 inhibition and glucokinase activation were evaluated using Mendelian randomization analysis, reporting the SD change per 1-mmol/L lower genetically predicted random plasma glucose via SGLT2 inhibition or glucokinase activation. Associations between acylcarnitines and heart failure were evaluated using Cox proportional hazards models, reporting the HR of heart failure per 1-SD higher of acylcarnitine levels.

**Supplementary Table 4. Associations of acylcarnitine scores with SGLT2 inhibition and heart failure**

| **Score** | **SGLT2 inhibition → Acylcarnitine score** | |  | **Acylcarnitine score→**  **Heart failure** | |
| --- | --- | --- | --- | --- | --- |
|  | **β (95% CI)** | ***P*** |  | **HR (95% CI)** | ***P*** |
| AC | -2.04 (-3.79, -0.29) | **0.023** |  | 0.97 (0.93, 0.99) | **0.045** |
| Short-chain AC | -0.71 (-1.45, 0.04) | 0.063 |  | 0.92 (0.86, 0.98) | **0.017** |
| Median-chain AC | -0.80 (-1.45, -0.16) | **0.015** |  | 0.93 (0.82, 1.05) | 0.223 |
| Long-chain AC | -0.43 (-0.97, 0.10) | 0.110 |  | 0.91 (0.82, 1.01) | 0.085 |

Unweighted AC scores were calculated by summing levels of AC with P < 0.1 for association with SGLT2 inhibition or heart failure. Associations between acylcarnitine scores with SGLT2 inhibition were evaluated using Mendelian randomization analysis, reporting the SD change per 1-mmol/L lower genetically predicted random plasma glucose via SGLT2 inhibition. Associations between acylcarnitine scores and heart failure were evaluated using Cox proportional hazards models, reporting the HR of heart failure per 1-SD higher of acylcarnitine levels.

**Supplementary Table 5. Associations of acylcarnitine scores with glucokinase activation and heart failure**

| **Score** | **Glucokinase activation → Acylcarnitine score** | |  | **Acylcarnitine score→**  **Heart failure** | |
| --- | --- | --- | --- | --- | --- |
|  | **β (95% CI)** | ***P*** |  | **HR (95% CI)** | ***P*** |
| AC | -1.47 (-3.06, 0.11) | 0.068 |  | 0.96 (0.93, 0.99) | **0.030** |
| Short-chain AC | -0.48 (-1.11, 0.15) | 0.137 |  | 0.90 (0.83, 0.98) | **0.019** |
| Median-chain AC | -0.35 (-0.77, 0.06) | 0.097 |  | 0.86 (0.74, 0.99) | **0.039** |
| Long-chain AC | -0.55 (-1.20, 0.09) | 0.091 |  | 0.93 (0.86, 1.01) | 0.073 |

Unweighted AC scores were calculated by summing levels of AC with P < 0.1 for association with glucokinase activation or heart failure. Associations between acylcarnitine scores with SGLT2 inhibition were evaluated using Mendelian randomization analysis, reporting the SD change per 1-mmol/L lower genetically predicted random plasma glucose via SGLT2 inhibition. Associations between acylcarnitine scores and heart failure were evaluated using Cox proportional hazards models, reporting the HR of heart failure per 1-SD higher of acylcarnitine levels.
